# Feasibility assessment for E-commerce: A data collection from developing country (Ethiopia)

**DOI:** 10.1016/j.mex.2022.101639

**Published:** 2022-02-20

**Authors:** Nagender Singh, Omprakash Sahu

**Affiliations:** a Department of Fashion and Apparel Engineering, TIT&S Bhiwani (Haryana), India; b Department of Chemical Engineering, UIE Chandigarh University, Mohali (Punjab), India

**Keywords:** E-commerce, Feasibility, ICT, FDI, Digital literacy, Ethiopia, ICT, Information and communication technologies, GDP, Gross domestic product, CSV, Comma-separated values, SEMs, Small and medium-sized enterprises

## Abstract

This methodology work has been made to investigate the factors which affect the feasibility and readiness of e-commerce in developing nations. An online and offline survey has been carried out to collect feedback from the banks, government bodies, consumers, and e-retailers all over Ethiopia and their responses have been analyzed through descriptive statistics by using Statistical Package for the Social Sciences software program. The results and finding give a clear understanding of the feasibility and readiness of e-commerce and reveals that the technological and organizational aspects, lack of Information and Communications Technology infrastructure, cross-country legal and regulatory differences, lack of digital literacy among consumers and businesses in terms of computer literacy, language barriers, lack of distribution system, lack of trust on e­commerce are the factors which affect the feasibility of e-commerce in Ethiopia. The feasibility can be improved by providing suitable information and communications technology infrastructure, improving cross-country regulatory differences, promoting ICTs in education targeting all levels of the educational system, facilitating foreign direct investment drive in ICTs, making a website attractive and user-friendly. The results of statistical analyses indicate that Ethiopia has significant potential for the feasibility of e-commerce.

• E-Commerce offers potential in the form of enhanced participation in the international value chain and public relations.
• The feasibility can be improved by providing suitable ICT infrastructure, improving cross-country regulatory differences.
• This research study reveals interesting facts about the potential and opportunity of e-commerce in Ethiopia.

## Graphical abstract

**Figure:**
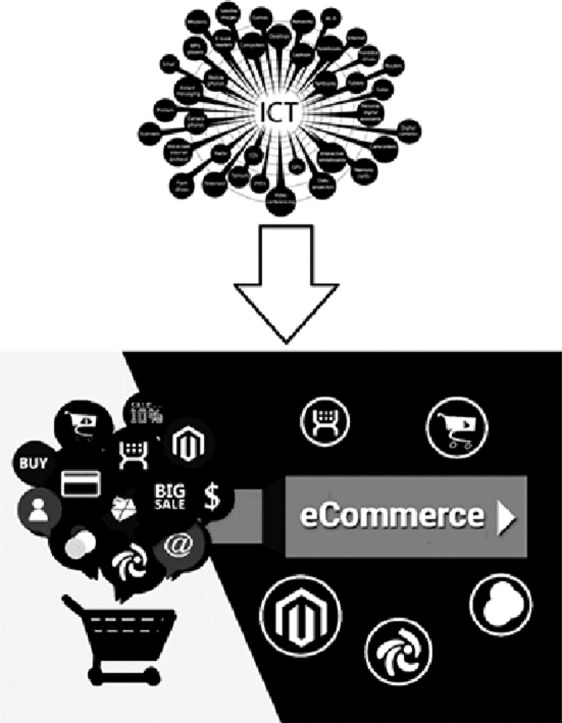

**Specifications table**

**Table:** 

Subject Area:	E-Commerce
More specific subject area:	Business; Finance; Management
Method name:	Statistical Analyses Method
Name and reference of original method:	NA
Resource availability:	NA
Direct submission or co-submission:	**Direct submission**

## Introduction

Over the last few decades, the world economy has dramatically changed due to advances in Information and Communication Technologies (ICTs) [1]. ICTs are changing economic and social activities and also providing challenges and lots of opportunities. ICT makes businesses more competitive, economies more productive and strengthens common people and organizations with knowledge and exposure [2]. This opened up new opportunities and perspectives with a number of applications. In today's world, ICT diffused into almost all corners of human activity [3]. This has led to the development of business processes and activities such as e-trade, e-commerce, and recently m-commerce, which imply the use of ICT tools and techniques inside and outside the environment in conducting day-to-day business process operations [4]. ITCs are fundamentally changing international trade, affecting business practices and introducing new business intermediaries such as e-commerce and e-banking. Governments play a critical role in the creation of an enabling policy environment to support these activities [5]. In Africa, e-commerce can offer new opportunities to export-oriented companies, especially small, medium and micro-enterprises. By using electronic and internet networks, SMMEs can source production inputs more efficiently by eliminating the intermediaries, reducing supply and distribution chains network, and effectively reducing business transaction costs [6].

E-commerce comprehends not only buying and selling goods over electronic networks, but also various business processes within individual organizations. Like e-commerce, e-business (electronic business) also has several different definitions and is used in many different contexts [7]. Commercial (e-tailing and e-banking) and non-commercial (e-government and e-health) both are the backgrounds for e-commerce. E-commerce offers increased convenience, lower costs of transactions, greater accessibility, price flexibility, increased consumer choices of interest by eliminating time and space constraints [7]. Ethiopia has registered with an average 10.9% annual growth over the past ten years and remarkable economic performance. This is double the Sub Sahara Africa and over this period it is triples the world average growths and has led to Ethiopia being rated as one of the fastest-growing economies in the world [8]. Ethiopia's GDP reached $ 115Billion with a per capita GDP of $650 by end of 20222. An agriculture farm, industry, and services sectors like e-commerce and information technology contributed 40%, 14%, and 46% to the GDP [9, 10]. This indicates that Ethiopia has the potential to introduce e-commerce in the service sector, agriculture, and industries.

This methodology initiative examines the primary aspects that influence e-commerce feasibility in Ethiopia. The purpose of this assessment is to provide a better understanding of the essential points where the Ethiopian government, banks, and e-retailers have to focus on and provide better facilities to their final consumer nationally and internationally for the growth of the country. This work explains the fundamentals of e-commerce and makes recommendations for future e-commerce development plans.

## Overview on information and communications technology

The shift towards e-commerce is already transforming the behavior of consumers and businesses. ICT applications and services role can be seen across the entire value chain of e-commerce. The e-commerce process can divide into four stages: information gathering, agreement, transaction, and delivery [11]. Information and communication technology is a set of theories and scientific-practical approaches and knowledge that facilitates the process, production, information management, and communication. The combination of three parts electronic integrated circuits and elements, information processing, and network requirements have led to the concept of information and communication technology [12]. The prosperity of electronic, telecommunication, and computer industries, gives information wave that carries human beings to a new information era. The new technologies provide important features i.e. volume of data and information which is generated, processed, and analyzed [13]. These kinds of features create a new technology called information technology which facilitates working with high volume data and exchange of information. The word information technology is used to support and optimize active information-based systems and facilitates the analysis of information efficiently [14].

Over the last decade, ICT has become an integral part of developing countries in development programs. The country faces a significantly great gap between interest in the ICTs and regulatory instruments and the policy developed by the government to enable the economic development of the country [15]. ICT has one of the major elements of a plan for accelerated and sustainable development to end poverty in Ethiopia [16]. E-commerce is the use of digital information processing technology and electronic communications networks in business transactions to create, transform, and redefine relationships among the organizations, between individuals and organizations [17]. E-commerce also refers to the electronic exchange of information pre-sales and the use of electronic communications for after-sales services. Different types of e-commerce are business-to-business (B2B); business-to-consumer (B2C); consumer-to-consumer (C2C); business-to-government (B2G); and mobile commerce (m-commerce) [18]. E-commerce is an integral part of ITC and its applications enable companies to communicate with their partners, suppliers, and consumers on the internet efficiently and effectively. The fundamental changes are required in business operational systems when the migration from traditional business to e-commerce is required [19]. One of the vital points in e-commerce is attracting customers' loyalty and trust which is essential from a potential and opportunity point of view. The continuous growth and development of successful e-commerce particularly depend upon consumers’ trust in e-commerce transactions [20].

There is no doubt that electronic commerce presents enormous opportunities for consumers and businesses (particularly for SMEs) in developing countries like Ethiopia [21]. The prediction is that with the right e-commerce policy framework and implementation strategy for rolling-out e-commerce programmers and initiatives. It is also necessary enabling and regulatory environment, the development and implementation of e-commerce will among other things make it possible for Ethiopia. To develop its economy, improve its export earnings and facilitate trade within the sub-region and with other countries on a global scale [22]. The importance of e-commerce and e-trade in developing Ethiopia's export-based cannot be under-estimates the contribution to economic growth and socio-economic development of the country. Ethiopian businesses could get benefit from trading their goods and services to prospective customers (governments, businesses, and consumers) within the country (local or domestic e-commerce), within the region (regional e-commerce), and on the global scale (global e-commerce) [23].

To assess the e-commerce potential in a country, it is useful to consider a simplified e-commerce transaction process. Several facilitating factors influence the scope for implementing e-commerce successfully such as online transactions i.e. e-payment, affordable internet access, processes for paying for goods and services during online purchase, and effective solutions for their delivery (electronically or physically) i.e. shipping [24]. The legal and regulatory framework, skills to implement e­ banking system, ICT infrastructure, cross-country regulatory differences, Digital literacy among consumers and businesses in terms of computer literacy, language barriers, Facilitating foreign direct investment (FDI) drive in ICTs, which also influence the extent to which enterprises and consumers are willing to transact online [25].

## Research methodology

The present assessment is based on applied research, due to the use of text analysis, and field methods such as questionnaires, the study can be considered as a descriptive statistics survey research. Conducting an online and offline survey in form of a questionnaire, seemed like the most appropriate tool for a survey, to be able to get a bigger picture of all the sector's opinions on the matter. The data have been collected via online and offline mode through questionnaires (https://docs.google.com/forms) because google forms are a perfect tool for creating online forms and surveys [26]. The form responses are automatically saved and can be easily exported from Google spreadsheet to other formats like PDF or CSV. To process data, the software SPSS has been used. The research data analysis has been done in descriptive statistics [27]. In the level, descriptive statistics, frequency, valid percentage, the cumulative percentage have been used to get accurate results. SPSS is capable of handling a large volume of data and can be able to perform all of the analyses covered in the text and much more. SPSS is commonly used in the Social Sciences and the business world [28]. The purpose of the questionnaire was to obtain feedback from the bank, government, e-retailers, and consumers all around Ethiopia. The questionnaire questions have been based on the searched literature review. Different types of questions have been included in the questionnaire. According to author Saunders, there are different kinds of questions that can be used, depending on requirements [29]. The questionnaire has 4 sections as per the data requirement i.e. banking sector, government, e-retailers, and consumers.

The banking section of the questionnaire has been used to collect data from banks to access the current scenario and viewpoint of managers towards e-commerce readiness; the other sections have been used to collect the feedback from government, e-retailers, and consumer's perceptions towards the feasibility of e-commerce in Ethiopia.

## Data analysis and results

In this section, the results of data analysis are examined and classified. The questionnaire consists of four sections as discussed in the research methodology.

### Section 1 (Questionnaire for banking sector)

The respondents of this section were 42 employees from Ethiopian Commercial Bank in Bahir Dar city Ethiopia.

• Technological point of view, which of the factors affects the feasibility of E­commerce in Ethiopia?

**Fig. 1:**
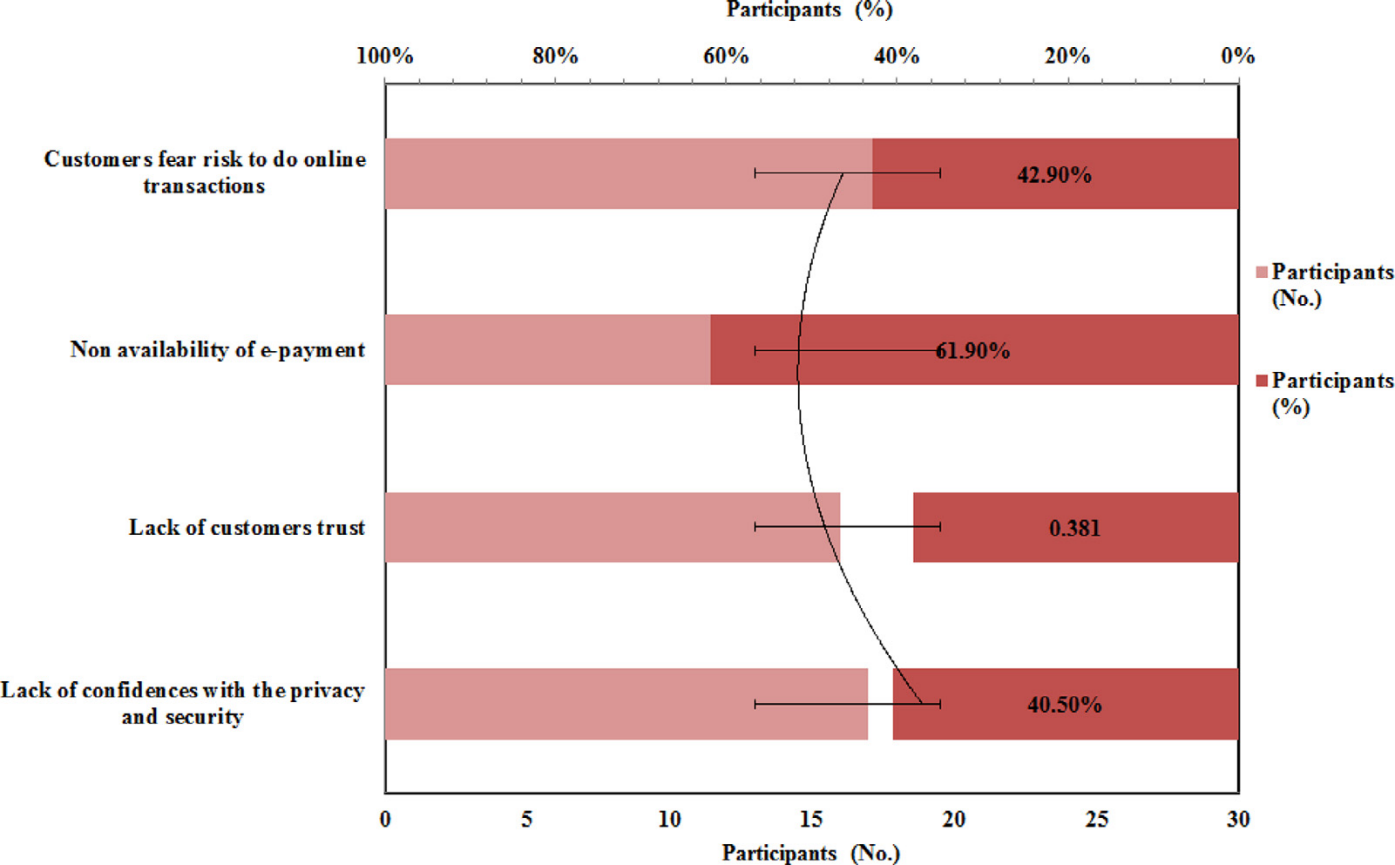
Technological factors influencing feasibility of e-commerce

Fig. 1 represents the feedback given by the respondent on technological factors influencing the feasibility of e-commerce. As shown in Fig. 1 when asking about the technological factors which affect the feasibility of e-commerce in Ethiopia, the majority of respondents 61.9 % respond that non-availability of an e-payment system for transactions is the major factor that could influence e-commerce potential in Ethiopia. This may be due to various fields and activities; essential requires the usage of the internet [30].

• What are the organizational factors that influence the implementation of a secure e­ payment system?

**Fig. 2:**
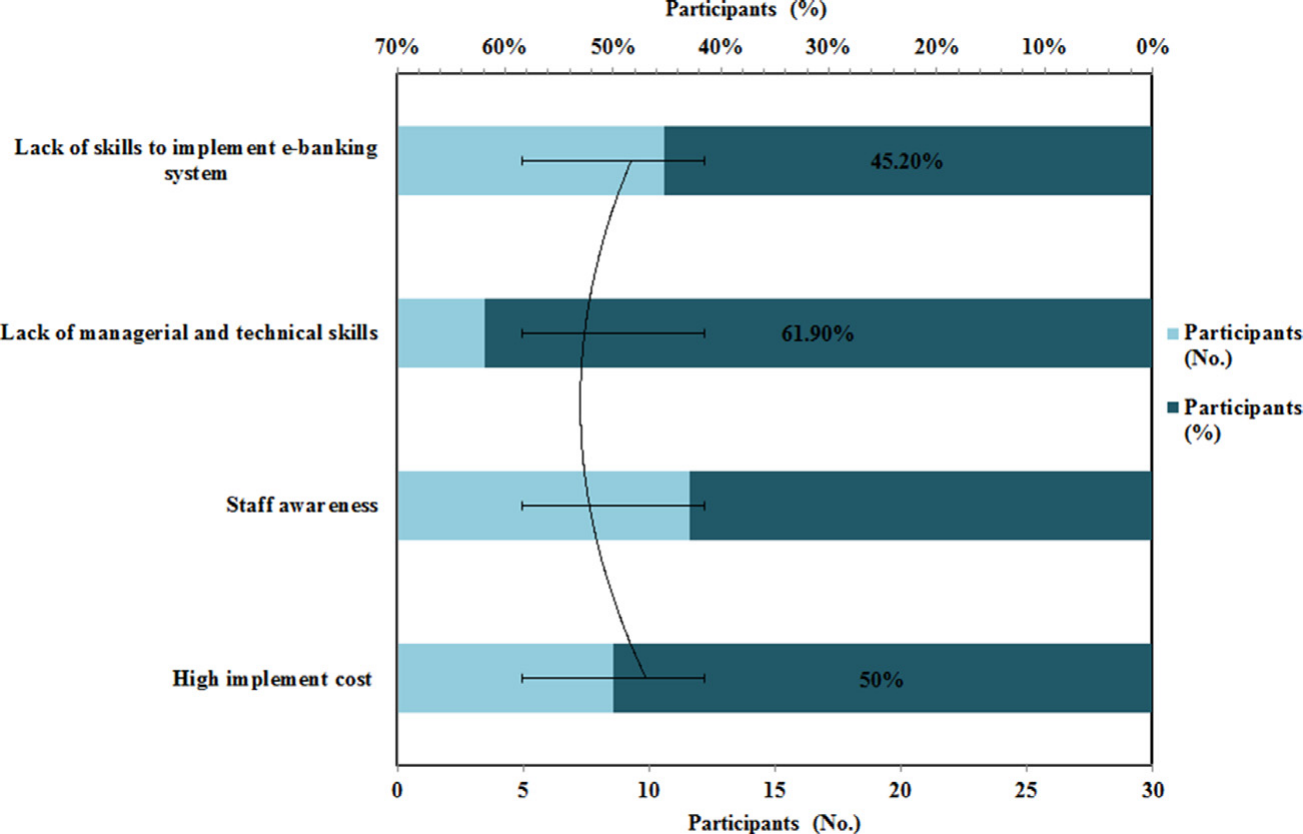
Organizational factors influencing implementation of secure e payment system

Fig. 2 represents the organizational factors influencing the implementation of a secure e­ payment system. It can be seen in Fig. 2, the graphical representations of respondents about organizational factors which influence the implementation of a secure e-payment system in the Commercial Ethiopian Bank. 61.9 % of respondents have been chosen that lack of managerial and technical skills is the main barrier in the implementation of secure e-payment systems i.e. online transactions. Managerial talents that relate to the technique of supervising and making organization choices and technical abilities are the skills and expertise required to do specialized activities in a certain industry [31].

• What are the challenges that banks are facing for e­ payment implementation?

**Fig. 3:**
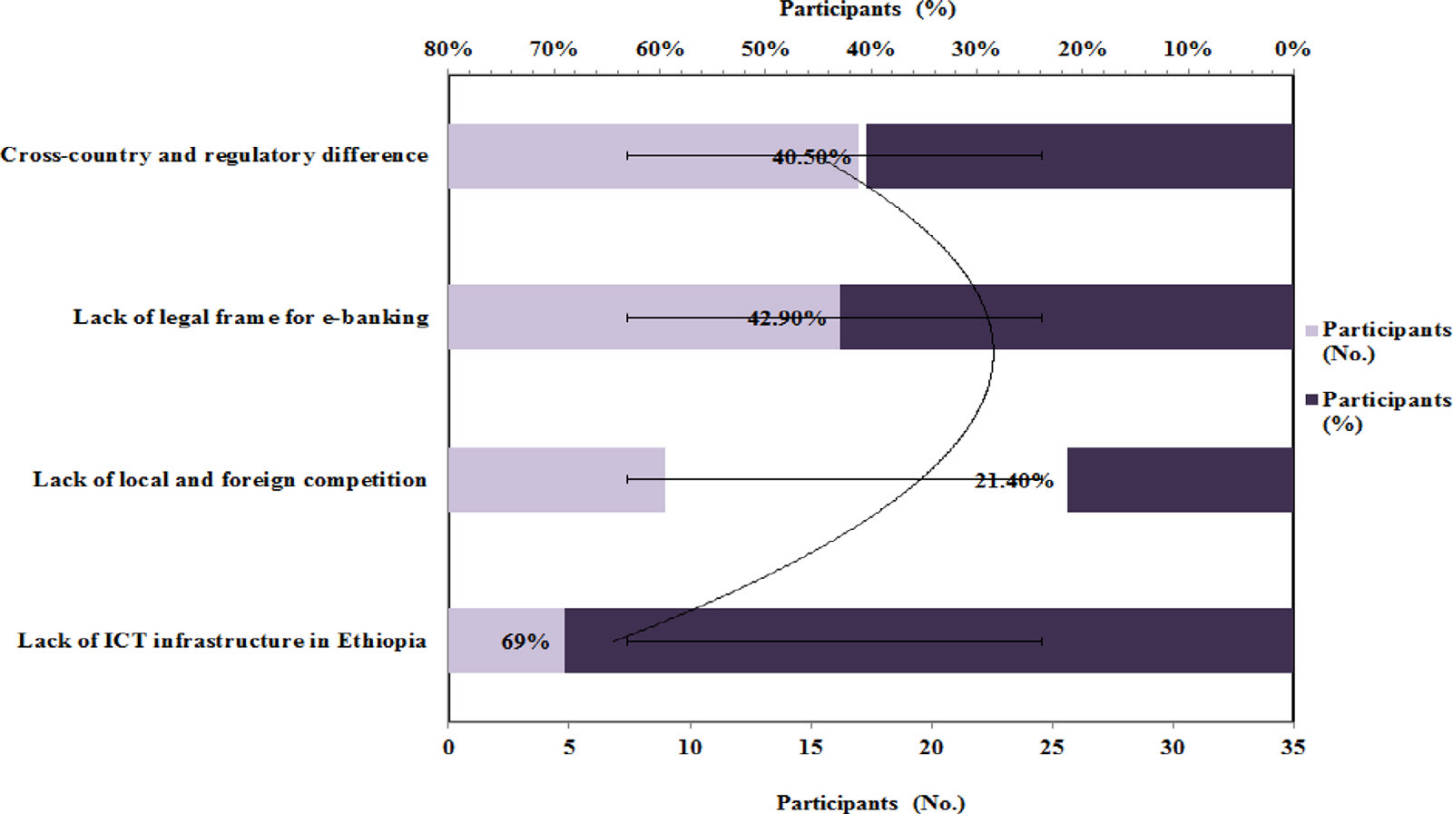
Challenges that bank are facing for e-payment implementation

Fig. 3 shows the challenges that banks are facing for e-payment implementation in Ethiopia. It shows 69 % of respondents chose lack of ICT infrastructure in Ethiopia as the main challenges that banks are facing for e-payment implementation and 42.9 % responded to lack of legal frame for e-banking is another challenge for e-payment implementation. Both the factors are really important for the feasibility of e-commerce in Ethiopia. Since, ICT Infrastructures included software, hardware, firmware, networks, and the company websites that are used for e-commerce in Ethiopia. Ethiopia's electronic banking system, according to economists, requires appropriate legal and regulatory frameworks to make the sector globally profitable. Experts also claimed that, despite its quick growth, the business is still in its development [32].

• In your opinion, what measures the government has to take to facilitate the e-commerce in Ethiopia?

**Fig. 4:**
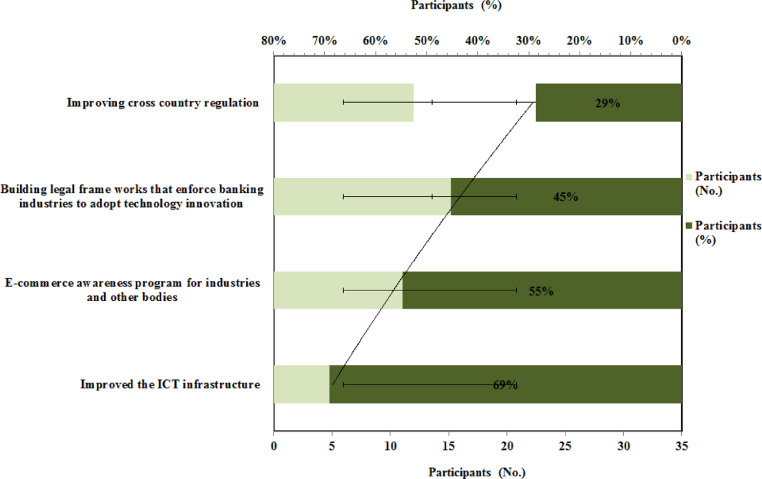
Respondents opinion and suggestions for the government

The graphical representation of the respondent's opinion and suggestion about the measures steps government have to take to facilitate the e-commerce in Ethiopia is presented in Fig. 4. It found that the majority of respondents i.e. 69 % suggest that government could facilitate the e-commerce program in Ethiopia by improving the ICT infrastructure in the country; in addition to that 54.8 % of respondents suggest that government has to do an e-commerce awareness program for industries and other bodies. Alibaba and the Ethiopian government reached a settlement to accelerate the e-commerce sector in Ethiopia. To foster a strong partnership, Alibaba will deliver 10-day training at Alibaba Business School in China to Ethiopian business owners. The program is aimed towards ambitious Ethiopian business executives and entrepreneurs who are committed to using digital technology to deliver their company and achieve long-term success. The program's goal is to teach attendees how to speed up their company's digital development and collaborate with other stakeholders to create success stories that will serve as building blocks for their indigenous governments and, finally, the digital economy as a whole [33].

### Section 2 (Questionnaire for government sector)

The online and offline respondents of this section were 28 government sector employees i.e. ethio-telecom, ITC ministry in Ethiopia.

• Do you think e­commerce will help improve the growth of a country?

The descriptive analysis of respondents' feedback in frequency, valid percentage, and the cumulative percentage is shown in Table 1. It was observed that most of the respondents 75 % agreed with the fact that, e-commerce can help improve the overall growth of the country. The development of e-commerce necessitates cross-border business and therefore contributes to international and national trade [34]. The entrance of information communication and technologies ICTs to allow consumers, entrepreneurs, and enterprises to buy and sell their products over the internet and electronic media cause evocation of the name electronic commerce. E-Commerce offers potential in the form of enhanced participation in the international value chain, increased market access and research, and improved internal and market efficiency, as well as lower transaction. It is enormously significant for the growth and development of the developing country [34].

• Challenges of government on e­commerce adoption in Ethiopia?

**Table 1:** Perception towards e-commerce for the growth of country

		Frequency	Percent	Valid Percent	Cumulative Percent
Valid	No	7	25.0	25.0	25.0
	Yes	21	75.0	75.0	100.0
	Total	28	100.0	100.0	-

**Fig. 5:**
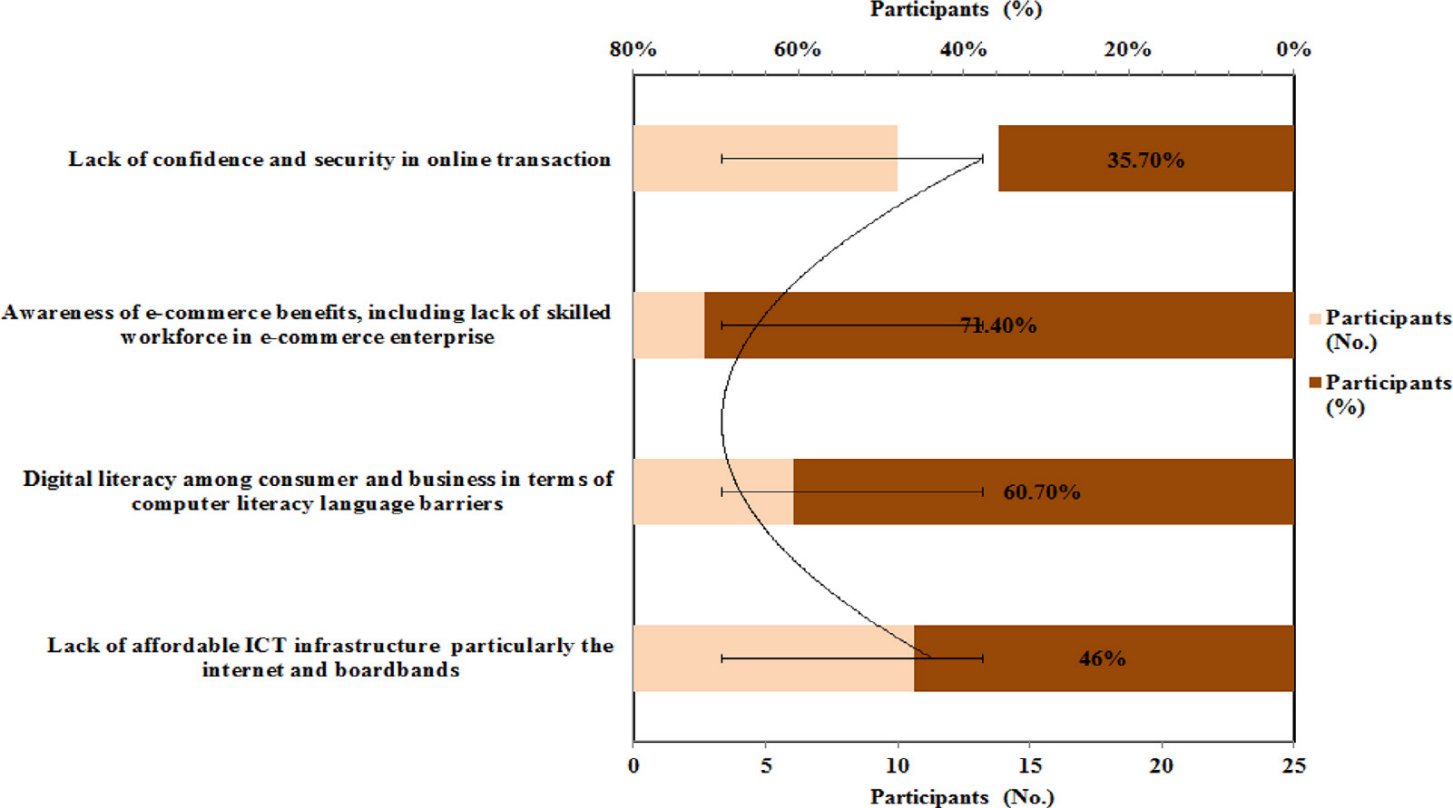
Challenges of government on ecommerce adoption in Ethiopia

The challenges of e-commerce adoption in Ethiopia are presented in Fig. 5. As shown in Fig. 5, when asking about challenges that the government is facing on e-commerce adoption in Ethiopia, majorities 71.4 % of respondents responds that awareness of e-commerce benefits, including lack of a skilled workforce in e-commerce enterprises, is the main challenge. The skilled workforce is generally defined by their education, expertise level and wages received. Now the government is advancing in enhancing education attainment levels. Government investment in vocational programmers and universities will improve the labor market over the long term [35]. The second response was 60.7 % that digital literacy and language barriers are other challenges that the government is facing on e-commerce adoption in Ethiopia.

• Do you agree the national ICT for development (ICT4D) five years action plan for Ethiopia [2006 – 2020] is helpful for the e-commerce sector?

The descriptive statistics of respondents about the national ICT for development (ICT4D) five years action plan as shown in Table 2. It can be identified that most of the respondents 50 % agreed that the national ICT for development (ICT4D) five years action plan for Ethiopia [2006 – 2020] is helpful for the e-commerce sector. Ethiopia's Growth and Transformation Plan (GTP), which is in its second five‑year phase, to help accelerate economic growth and achieve the developmental goals of becoming a middle‑income country by 2025 [36, 37].

• How government can improve the potential of e-commerce in Ethiopia?

**Table 2:** Response about national ICT for development (ICT4D) five years action plan for Ethiopia

		Frequency	Percent	Valid Percent	Cumulative Percent
Valid	Agree	14	50.0	50.0	50.0
	Disagree	3	10.7	10.7	60.7
	Neutral	4	14.3	14.3	75.0
	Strongly Agree	6	21.4	21.4	96.4
	Strongly Disagree	1	3.6	3.6	100.0
	Total	28	100.0	100.0	-

**Fig. 6:**
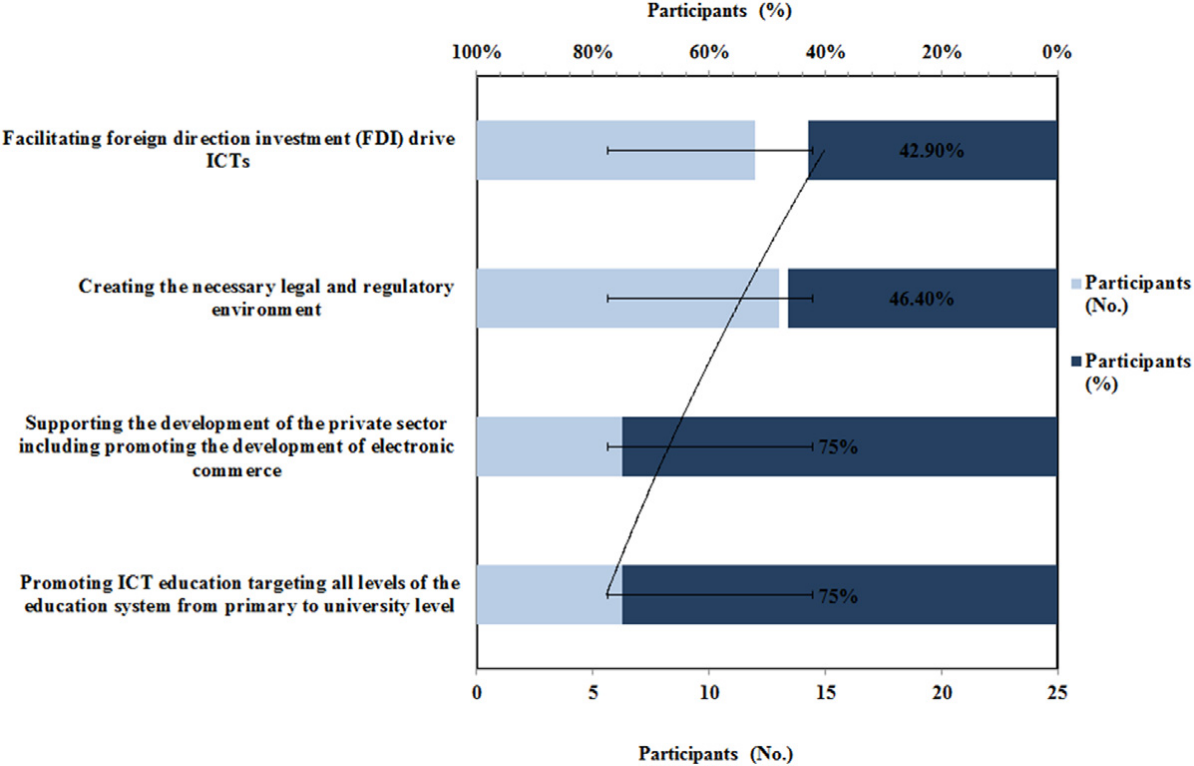
Responses on improvement of e-commerce potential in Ethiopia

The respondent's feedback on the improvement of e-commerce potential in Ethiopia is represented in Fig. 6. As shown (Fig. 6), the majority of respondents 75 % responded that government can improve the e-commerce potential by promoting ICTs in education targeting all levels of the educational system and supporting the development of the private sector including promoting the development of e-commerce. Next to housing or public services and permissible environment, the growth of society is provided through education that strengthens all aspects in the ITC sector [38].

### Section 3 (Questionnaire for e-retailers)

The online respondents of this section were 34 e-retailers all around Ethiopia.

• Challenges for e-commerce in Ethiopia?

**Fig. 7:**
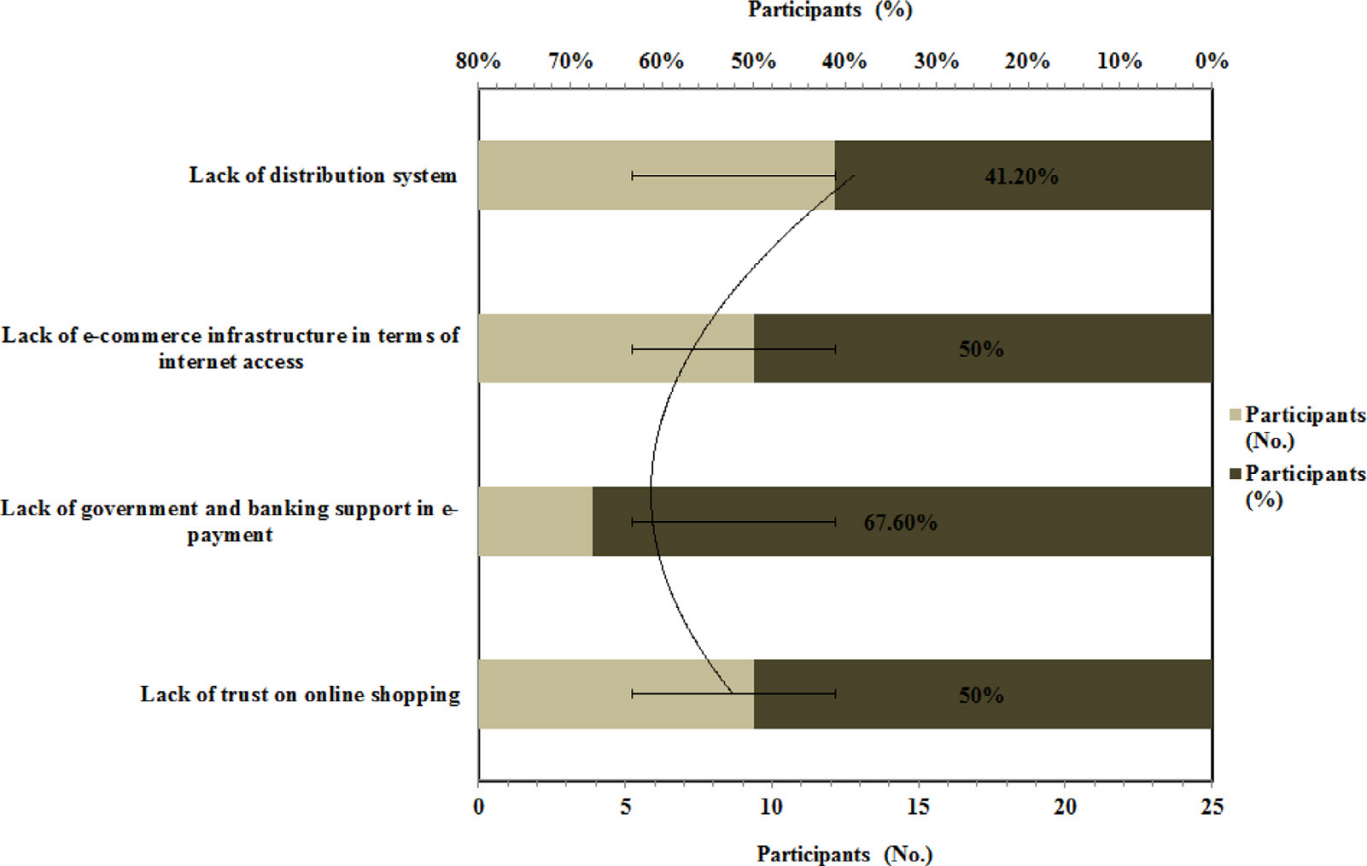
Challenges for e-commerce in Ethiopia

The challenge for e-commerce in Ethiopia is shown in Fig. 7. It can be seen a majority of respondents 67.6 % responded that lack of government and banking support in e-payment is the main challenge for e-commerce in Ethiopia. In comparison to the majority of the world, Ethiopia's financial sector is one of the least developed. Cash is still the most common form of payment in Ethiopia, and electronic banking is barely understood, less alone utilized for financial transactions [39, 40]..

• Does Ethiopia have e-commerce potential and opportunities?

As shown in Table 3, the descriptive statistics of respondents' feedback about e-commerce potential and opportunities in Ethiopia. It has been noticed that most of the people 41.2 % agreed that Ethiopia has enough potential and lots of e-commerce opportunities. Ethiopia was slow to adopt and apply these technologies at first, but it is presently trying to do so by developing legal and technological frameworks as well as infrastructure. The rapid growth of broadband customers in Ethiopia is a benefit for the digital commercial concept. Many company industries, such as travel, clothes, and electronics, can benefit from E-commerce in various ways [41, 42].

• According to you what are the main barriers to purchasing clothing online?

**Table 3:** Ethiopia e-commerce potential and opportunities

		Frequency	Percent	Valid Percent	Cumulative Percent
Valid	Agree	14	41.2	41.2	41.2
	Disagree	9	26.5	26.5	67.6
	Neutral	2	5.9	5.9	73.5
	Strongly Agree	4	11.8	11.8	85.3
	Strongly Disagree	5	14.7	14.7	100.0
	Total	34	100.0	100.0	-

**Fig. 8:**
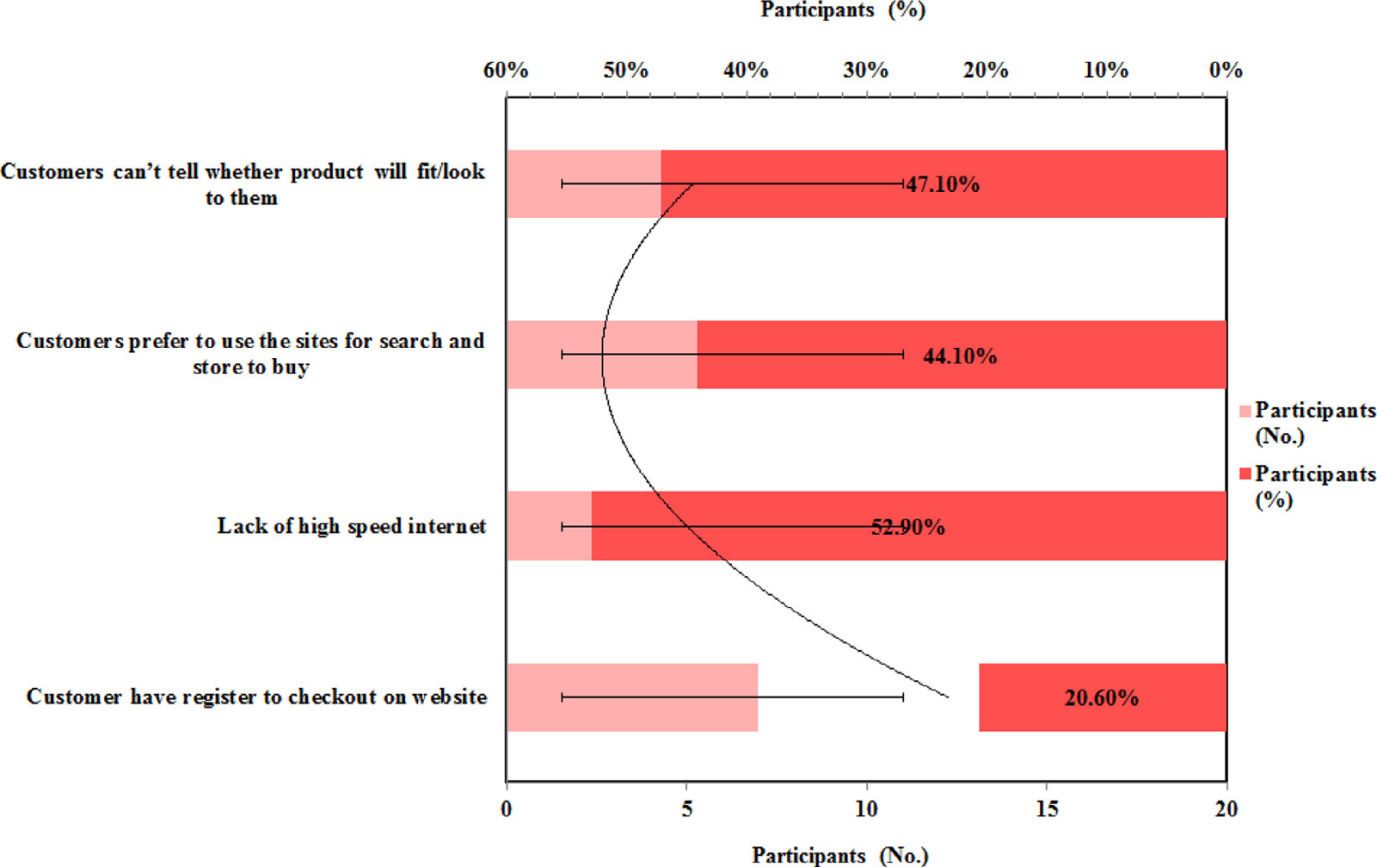
Main barriers to purchase clothing online

The respondent's feedback for the main barriers to purchasing clothing online is represented in Fig. 8. It indicates that the majority of respondents 52 % felt that lack of high-speed internet is the main barrier to making the purchase online and 47.1 % responds that lack of customer feedback is another barrier. As per the survey, Ethiopia is ranked 116th out of 121 nations in terms of network readiness. The rating is based on a country's basic level of ICT, which includes factors such as communications infrastructure and cost, as well as how companies utilize ICT and participate in the digital industry. Ethiopia is classified as a low-income country in this ranking [43, 44].

• How to improve conversion rates on the websites?

**Fig. 9:**
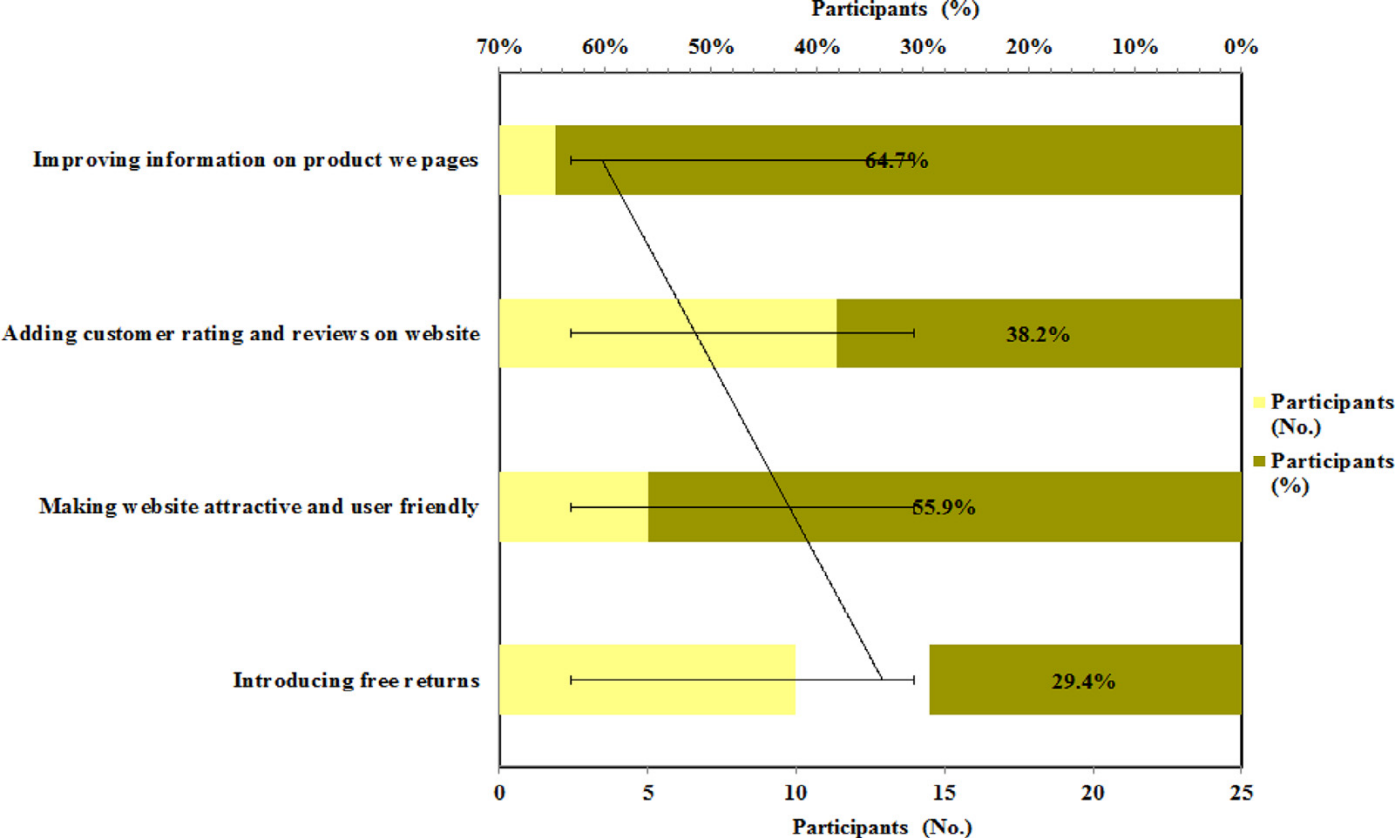
Improvement of conversion rates on the website

The suggestions given by the respondents on the improvement of conversion rates on the websites represent in Fig. 9. It found that the majority of people 64.7 % responds that improving the information of the product on product web pages can be helpful to improve conversion rates on the websites. The overall engagement percentage of e-commerce sites is 2.86%, according to the most recent survey and research in 2020. The overall e-Commerce webpage converting rate in the United States is 2.63%, whereas the global average is 4.31 [45]. Conversion rate data are some of the most secure types of data on the internet. Such confidentiality should be expected by the company. The concept of sharing the performance of their website with rivals does not appeal to website owners. While several tools exist to predict the visitor numbers a website receives, only a few software solutions exist to evaluate the conversion rate of any website [46].

### Section 4 (Questionnaire for consumers)

The online and offline respondents of this section were 192 consumers from all over Ethiopia.

• Do you have trust in e-commerce?

The descriptive statistics of respondents about the trust in e-commerce are mentioned in Table 4. As trusting is an important ingredient when talking about technology and innovations as well as essential to assess the potential of e-commerce in any country. As shown in Table 4, approximately 62.5 % of respondents were agreeing that they have trust in e-commerce, this shows consumers are willing to participate in e-commerce. African countries are experiencing great growth in online purchasing, and e-commerce companies are interacting with customers to improve their performance [47]. Ethiopia is rapidly expanding and taking steps to enhance its E-commerce performance. Around the year 2018, Ethiopia launched its internet business. The present condition of Ecommerce in Ethiopia is that small businesses are not developing due to a lack of infrastructure, financial capacity, and labor, as well as the usage of ICT, to benefit businesses [48].

• How frequently do you purchase products online?

**Table 4:** Trust on e-commerce

		Frequency	Percent	Valid Percent	Cumulative Percent
Valid	Agree	120	62.5	62.5	62.5
	Disagree	18	9.4	9.4	71.9
	Neutral	28	14.6	14.6	86.5
	Strongly Agree	24	12.5	12.5	99.0
	Strongly Disagree	2	1.0	1.0	100.0
	Total	192	100.0	100.0	-

The descriptive analysis of consumers' feedback on the frequency of online purchases is mentioned in Table 5. It shows in Table 5 that the majorities 85.4 % of respondents don't have an online purchasing experience; this indicates that consumers are afraid to purchase their product online. According to a recent IPSOS poll conducted by the Center for International Governance Innovation in the years 2016-2017, approximately 51% of global people lack faith in online companies with internet access because of privacy and security concerns [49]. This is a terrible position for online shops that are trying to grow their personalization business. In the study on bankers' perceptions, it was discovered that while utilizing electronic banking, bankers perceive to reduce time and decrease difficulties [50].

• Why do you prefer to purchase products from e-commerce websites?

**Table 5:** Consumer online purchase frequency

		Frequency	Percent	Valid Percent	Cumulative Percent
Valid	Never	164	85.4	85.4	85.4
	Once in a month	4	2.1	2.1	87.5
	Once in year	16	8.3	8.3	95.8
	Once or twice in a 5-6 months	8	4.2	4.2	100.0
	Total	192	100.0	100.0	-

The descriptive statistics of respondents' feedback when asking about the preference for the purchased product on online shopping websites are shown in Table 6. It is shown that the majority of respondents respond by 45.3 % that easy to assess and order the product online is the reason behind consumers' online shopping preference. The availability of various payment options in a given nation is the most important need for payment collecting. In terms of credit cards, Ethiopian banks would not provide them, and national banking firms have just lately begun to use main online transactions via cellphone and card internet banking [51]. To get a digital payment facility, it must have access to the internet to make online purchases effective. Even if it has a tiny margin in a company, dishonest charges, chargebacks, and so on, once you are paid by a banking website, you find yourself surrendering a big portion of your earnings (maybe at least 4% or more) [52]. The merchants are then held accountable and liable inside the business model. Cash payment (COD) is a payment method that may be used to start an E-commerce business in Ethiopia. The security and confidentiality of nonpublic and commercial information transmitted through the internet are not guaranteed. Secure payment must be implemented for an online business to grow [53].

• If you do not purchase products through e-commerce why?

**Table 6:** Consumer preference to purchase product from e-commerce websites.

		Frequency	Percent	Valid Percent	Cumulative Percent
Valid	Availability of long range of products	10	5.2	5.2	5.2
	Convenient, Flexible and time saving	87	45.3	45.3	50.5
	Easy to assess and order	51	26.6	26.6	77.1
	Price flexibility	44	22.9	22.9	100.0
	Total	192	100.0	100.0	-

**Fig. 10:**
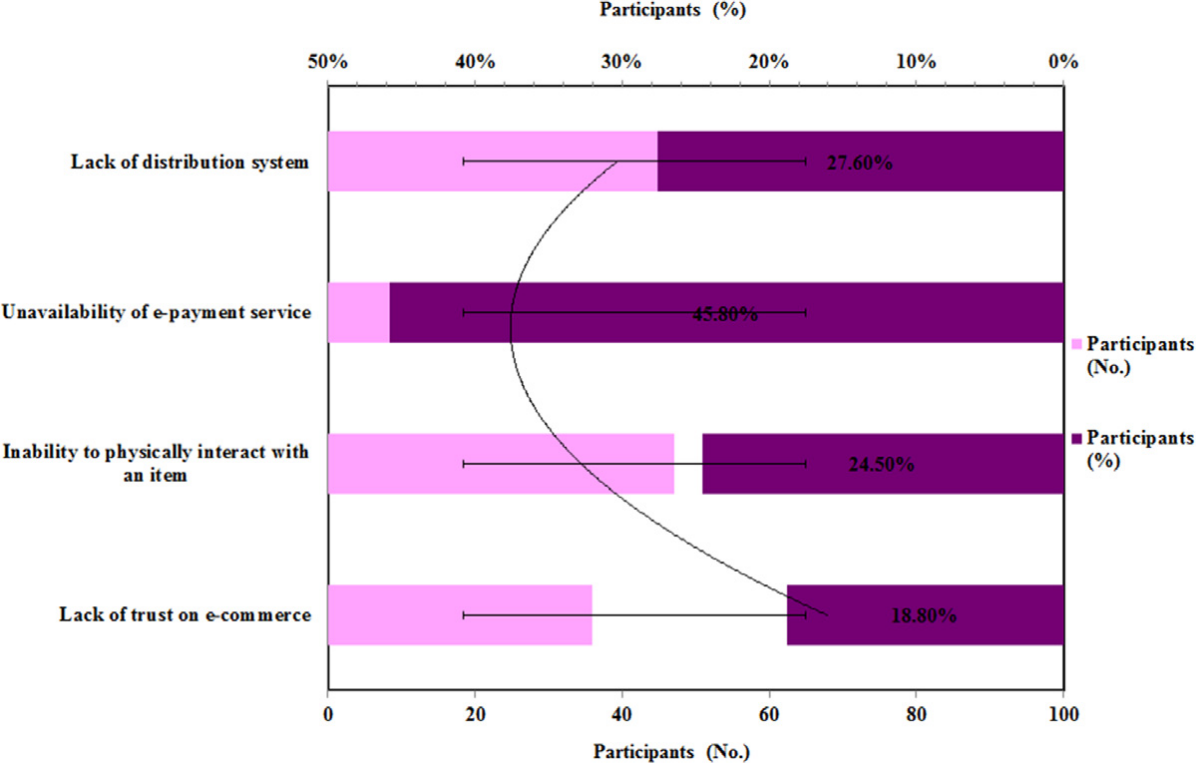
Consumers feedback on purchase of product through e-commerce

The consumer's feedback on the purchase of a product through e-commerce is represented in Fig. 10. The graphical representation shows that most of the consumers respond 45.8 % that if they are not purchasing the products through e-commerce because of unavailability of e-payment system and 27.6 % believe that lack of distribution system is another reason not to purchase products through e-commerce. The slow growth of E-commerce in African countries, particularly Ethiopia, is due to some infrastructure barriers. One of the impediments is the lack of information technology training for employees to improve their ability to utilize the internet. The proper computer knowledge and expertise of employees are the other. As a result, a strategic strategy is required [54]. Some of the obstacles include a lack of IT systems, a lack of understanding among individuals, consumer behavior, and, perhaps most importantly, the lack of a legislative structure. Ethiopia's status as a landlocked developing nation (LLDC) is also a hindrance to its E-commerce development [55].

## Conclusion

This research study reveals interesting facts about the potential and opportunity of e-commerce in Ethiopia. There is no doubt that Ethiopia has significant potential and opportunities for e-commerce. From the literature and results, it can be concluded that there is no significant impact of the national ICT for development (ICT4D) five years action plan for Ethiopia on e-commerce and e-trades sectors. This study also shows that Ethiopia doesn't have proper ICT infrastructure, e-payment, and distribution system i.e. shipping, managerial and technical skills for implementation of e-payment in the bank, awareness of e-commerce benefits; including lack of a skilled workforce in e-commerce enterprises and digital literacy among consumers and businesses in terms of computer literacy, language barriers and high-speed internet. This study concludes that the feasibility of e-commerce in Ethiopia is likely to be influenced by building legal frameworks that enforce banking industries to adopt technology innovation, improving cross-country regulatory differences, promoting ICTs in education targeting all levels of the educational system from school to university level, supporting the development of the private sector including promoting the development of electronic commerce, facilitating foreign direct investment (FDI) drive in ICTs.

## Declaration of Competing Interest

An Author Agreement is a statement to certify that all authors have seen and approved the final version of the manuscript being submitted. They warrant that the article is the authors' original work, hasn't received prior publication, and isn't under consideration for publication elsewhere.
